# Disparities in Patient-Reported Interest in Web-Based Patient Portals: Survey at an Urban Academic Safety-Net Hospital

**DOI:** 10.2196/11421

**Published:** 2019-03-26

**Authors:** Shobha Sadasivaiah, Courtney R Lyles, Stephen Kiyoi, Piera Wong, Neda Ratanawongsa

**Affiliations:** 1 Zuckerberg San Francisco General Hospital and Trauma Center University of California San Francisco San Francisco, CA United States; 2 Zuckerberg San Francisco General Hospital and Trauma Center UCSF Center for Vulnerable Populations University of California San Francisco San Francisco, CA United States; 3 Zuckerberg San Francisco General Hospital and Trauma Center San Francisco Department of Public Health San Francisco, CA United States

**Keywords:** patient portals, vulnerable populations, health care disparities, safety net hospitals, health information technology

## Abstract

**Background:**

Offering hospitalized patients’ enrollment into a health system’s patient portal may improve patient experience and engagement throughout the care continuum, especially across care transitions, but this process is less studied than portal engagement in the ambulatory setting. Patient portal disparities exist and may lead to differences in access or outcomes. As such, it is important to study upstream factors in a typical hospital workflow that could lead to those disparities in safety-net settings.

**Objective:**

The objective of this study was to evaluate sociodemographic characteristics associated with interest in a health care system’s portal among hospitalized patients and reasons for no interest.

**Methods:**

Nurses assessed interest in a Web-based patient portal, expressed by the patient as “yes” or “no,” as part of the admission nursing assessment among patients at an academic urban safety-net hospital and recorded responses in the electronic health record (EHR), including reasons for no interest. We extracted patient responses from the EHR.

**Results:**

Among 23,994 hospitalizations over a 2-year period, 35.90% (8614/ 23,994) reported an interest in a Web-based portal. Reasons for *no* interest included the following: *not interested/other reason* 41.68% (6410/15,380), *no ability to use/access computers/internet* 29.59% (4551/15,380), *doesn’t speak English* 11.15% (1715/15,380), *physically or mentally unable* 8.70% (1338/15,380), *does not want to say* 8.70% (1338/15,380), *security concerns* 0.03% (4/15,380), and *not useful* 0.16% (24/15,380). Among the 16,507 unique patients included in this sample, portal interest was lower in older, African American, non-English speaking, and homeless patient populations.

**Conclusions:**

In a safety-net system, patient interest at the time of hospitalization in a Web-based enterprise portal—a required step before enrollment—is low with significant disparities by sociodemographic characteristics. To avoid worsening the digital divide, new strategies are needed and should be embedded within routine workflows to engage vulnerable safety-net patients in the use of Web-based health technologies.

## Introduction

### Background

Patient portals are websites that offer access to personal health information on the Web, such as test results, medical histories, immunization histories, and secure messaging with providers. Health care systems—leveraging federal incentives for meaningful use of electronic health records (EHRs)—are promoting patient portals to improve quality and safety, engagement, and care coordination with a goal of improved patient outcomes. Although the evidence is mixed about the benefits to health outcomes [1], portals have been associated with improvement in patients’ diabetes medication adherence [2], understanding of medical conditions [3], and retention in a health care system [4].

Demographic factors such as age and education affect patient interest and use of patient portals [5]. Ambulatory and inpatient research suggests racial and ethnic disparities in portal use [6,7]. However, early research shows that diverse patients from safety-net health care settings have high interest in portal features such as electronic communication with providers [8], suggesting an opportunity for intervention.

Safety-net health care systems, systems that care for vulnerable, uninsured, or predominantly Medicaid patient populations, may face additional challenges engaging patients in their portals. For patients in safety-net settings, health care systems must be able to assess interest in enrollment, including frequently explaining to patients what a portal is and how portals may be useful for their care across the care continuum. Although portals have traditionally been studied across ambulatory settings [6], enrollment in a health care organization’s portal during a hospital admission represents an opportunity to engage high-risk patients in their health. By providing patients information about their hospital course and recommendations for treatment and follow-up care, a health care system portal may help improve a patient’s postdischarge self-care, transitions to ambulatory care, and ultimately improve health outcomes.

### Objectives

No study has systematically assessed interest in a health network’s portal within a typical hospital workflow among hospitalized urban safety-net patients. This study examined the interest in an enterprise-wide Web-based portal reported by hospitalized patients, how interest varied by sociodemographic characteristics, and the barriers among those declining portal interest.

## Methods

### Overview

This cross-sectional study evaluated hospitalized patients’ interest in enrolling in a health care system’s portal at an urban academic safety-net hospital. The patient portal offered information about the hospitalization (hospital after-visit summary with discharge instructions, summary of hospital course, and test results), as well as ambulatory patient portal content (primary and specialty care after-visit summaries, medications, allergies, problem lists, test results, and secure messaging with primary care providers). Eligible patients included adults (aged 18 years and over) admitted to acute medical, surgical, or gynecologic obstetric units over a 2-year period (September 2015-September 2017). This sample excludes patients under 18 years of age, admitted to skilled nursing or psychiatric units or reporting existing portal accounts.

### Patient Assessment

Nursing leadership provided universal education to bedside nurses about the content and value of the patient portal and how to assess interest using an EHR-built admission assessment question stem (*Are you interested in learning more about or signing up for our free, secure website to view your health record online? The website gives you access to your lab results, medication lists, discharge instructions, and other information from your stay*). Nurses could record patient interest as *yes* or *no*, followed by a structured drop-down menu based on existing literature about portal barriers [9,10]: *no ability to use/access computers/internet*, *doesn’t speak English*, *physically or mentally unable*, *not interested/other reason*, *doesn’t way to say*, or nurses could write over a free- text reason. By incorporating this assessment into routine clinical operations, nursing leadership standardized the verbiage of the assessment, but nurses could individualize any clarifying questions or follow-up counseling about the portal. Nurses did not offer specific technology or portal demonstrations.

The data for this analysis were abstracted from the EHR nursing assessments as well as the EHR sociodemographic section (age, gender, race/ethnicity, language, insurance type, and homelessness).

### Analysis

The primary outcome was the proportion of patients with portal interest recorded as *yes* during at least one admission. As some patients may change their responses in subsequent admissions, we also calculated the proportion of portal interest expressed at the hospitalization level and calculated the proportion of reasons for *No* interest during each hospitalization, coding free-text reasons into the existing or separate categories.

We then used logistic regression with measured sociodemographic covariates to calculate the unadjusted and adjusted odds of portal interest during at least one admission. Statistical analysis was performed using Stata version 15.1 (StataCorp). The University of California San Francisco Institutional Review Board approved this study.

## Results

### Demographics

Among 16,507 unique patients, the average age was 53 years (SD 19.17): 42.16% (6960/16,507) were women, 26.43% (4362/16,507) white, 28.02% (4625/16,507) Hispanic, 18.56% (3063/16,507) African American, 19.06% (3147/16,507) Asian, 7.93% (1309/16,507) other. Furthermore, 49.35% (8146/16,507) preferred a non-English language--10.67% (1761/16,507) Spanish and 5.40% (892/16,507) Chinese. 80.50% (13,288/16,507) had Medicaid or Medicare, and 14.17% (2339/16,507) reported homelessness. There was an average of 1.4 admissions per patient in this cohort during the period studied (see Table 1).

### Reasons for Lack of Portal Interest

When analyzed across 23,995 admissions, patients reported interest in the portal during 35.90% (8614/ 23,994) of admissions. The most commonly documented reasons for *no* were the following: *not interested* 41.68% (6410/15,380), *no ability to use/access computers/internet* 29.59% (4551/15,380), *doesn’t speak English* 11.15% (1715/15,380) , *physically or mentally unable* 8.70% (1338/15,380), *does not want to say* 8.70% (1338/15,380), *security concerns* 0.03% (4/15,380), *not useful* 0.16% (24/15,380).

**Table 1:** Demographic characteristics of adults asked about portal interest during admissions to an urban academic safety-net hospital between September 2015 and September 2017.

Patient Characteristics (N=16,507)		Statistics
Age (years), mean (SD)		53.06 (19.17)
Women, n (%)		6960 (42.16)
**Self-reported race/ethnicity, n (%)**		
	White	4362 (26.43)
	Hispanic	4625 (28.02)
	African American/black	3063 (18.56)
	Asian	3147 (19.06)
	Native Hawaiian/Pacific Islander	222 (1.34)
	American Indian/Alaskan Native	120 (0.73)
	Other/mixed	967 (5.86)
**Primary language, n (%)**		
	English	8361 (50.65)
	Spanish	1761 (10.67)
	Cantonese, Mandarin, or Toishanese	892 (5.40)
	Other Asian languages, n (%)	208 (1.26)
	Other, n (%)	5285 (32.02)
**Insurance, n (%)**		
	Medicaid or county health plan for uninsured^a^	8278 (50.15)
	Medicare	5010 (30.35)
	Commercial insurance	1300 (7.88)
	Other insurance	1919 (11.63)
**Homeless status**		
	Yes^b^, n (%)	2339 (14.17)
	No, n (%)	12478 (75.59)
	Unknown, n (%)	1690 (10.24)
Admissions, mean (range)		1.4 (1-33)

^a^These health care access programs (administered by the county Medicaid managed care organization) provide medical services for uninsured county residents (aged 18-64 years) or adults who provide county-supported in-home support services.

^b^Patients reported *yes* during at least one admission.

### 

### 

**Figure 1:**
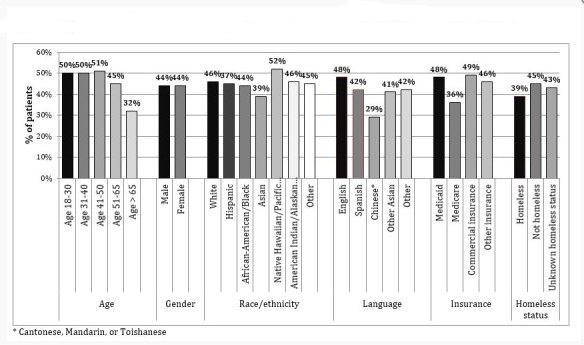
Patient interest in an online healthcare system portal during at least one admission to an academic safety net hospital (n=16507 patients, September 2015-September 2017).

### Interest by Sociodemographic Characteristic

However, 44% of unique patients reported interest in the patient portal during at least one admission. Figure 1 shows unadjusted proportions of interest in the portal during at least one admission, stratified across sociodemographic characteristics. In unadjusted analyses (Table 2), the odds of expressing interest in the Web-based portal were significantly lower for patients in the 50 to 65 years age group (adjusted odds ratio [AOR] 0.82, 95% CI 0.74-0.90 vs 18-29 age group), the greater than 65 years of age group (AOR 0.47, 95% CI 0.42-0.52 vs 18-29 age group), Asians (AOR 0.76, 95% CI 0.69-0.84 vs white), Spanish-speaking (AOR 0.78, 95% CI 0.70-0.86 vs English), Chinese-speaking (AOR 0.46, 95% CI 0.39-0.53 vs English), patients on Medicare (AOR 0.60, 95% CI 0.56-0.65 vs Medicaid), and patients reporting homelessness (AOR 0.77 95% CI 0.70-0.84 vs not homeless).

In adjusted models (Table 2), older age (AOR 0.85, 95% CI 0.77-0.95 for >50 and AOR 0.50, 95% CI 0.44-0.57 for >65 vs 18-29), African American race (AOR 0.87, 95% CI 0.79-0.96 vs white), Spanish or Chinese languages (AOR 0.69, 95% CI 0.61-0.78 and AOR 0.54, 95% CI 0.46-0.65 vs English), and homelessness (AOR 0.60, 95% CI 0.54-0.66 vs housed) were significantly associated with *No* portal interest during any hospitalization.

**Table 2:** Odds of expressing interest in a patient portal among patients at an urban academic safety-net hospital (n=16,507 unique patients).

Sociodemographic characteristic		Unadjusted odds ratio	*P* value	Adjusted odds ratio^a^	*P* value
**Gender**					
	Male	Ref	—^b^	Ref^c^	—
	Female	1.01	0.85	1.04	0.25
**Age (years)**					
	18-29	Ref	—	Ref	—
	30-39	0.99	0.91	1.01	0.9
	40-49	1.04	0.49	1.09	0.15
	50-65	0.82	<.001	0.85	0.003
	>65	0.47	<.001	0.5	<.001
**Race or ethnicity**					
	White	Ref	—	Ref	—
	Hispanic	0.98	0.65	0.93	0.15
	African American	0.95	0.24	0.87	0.01
	Asian	0.76	<.001	0.91	0.1
	Native Hawaiian/Pacific Islander	1.31	0.05	1.09	0.52
	American Indian/Alaskan Native	1.01	0.95	0.94	0.74
	Other	1	0.96	0.92	0.24
**Language**					
	English	Ref	—	Ref	—
	Spanish	0.78	<.001	0.69	<.001
	Cantonese, Mandarin, or Toishanese	0.46	<.001	0.54	<.001
	Other Asian	0.77	0.07	0.9	0.47
	Other	0.78	<.001	0.78	<.001
**Insurance**					
	Medicaid	Ref	—	Ref	—
	Medicare	0.6	<.001	0.91	0.07
	Commercial insurance	1.03	0.64	0.94	0.34
	Other insurance	0.94	0.2	0.98	0.71
**Homeless status**					
	Not homeless	Ref	—	Ref	—
	Homeless	0.77	<.001	0.6	<.001
	Unknown homeless status	0.91	0.07	0.85	0.003

^a^Logistic regression adjusted for all the variable listed in the table.

^b^Not applicable.

^c^Ref: reference value.

## Discussion

### Conclusions

Fewer than half of the patients admitted to a safety-net hospital were documented by nurses as interested in the patient portal during at least one of their admissions to a safety-net hospital, with significantly lower odds of portal interest among older, nonwhite, non-English-speaking, and homeless patients. This is the first attempt at universally quantifying how many hospitalized patients are interested in a health care system’s portal and highlighting the prevalence of patient barriers to portal engagement within a safety-net setting.

A notable finding from this analysis was that the proportion of hospitalizations during which patients expressed portal interest (31%) was lower than the proportion of patients expressing portal interest during at least one hospitalization (44%), a finding that remained true across sociodemographic categories. This finding suggests that offering the patient portal to a patient who has previously declined may be useful for subsequent engagement. Organizational, technical, or workflow protocols that fail to offer portal enrollment to patients who previously declined may exacerbate disparities. In safety-net systems, the simple intervention of standardizing offers with every hospitalization may be an important tool for engaging patients in these technologies.

Most hospitals—particularly safety-net hospitals with limited resources—may rely on their existing staff to engage patients in their portal. This paper reveals what may naturally happen in a typical workflow where engagement happens universally as a required part of admission. A strength of this study is that the nursing staff were essential partners and allies for portal promotion given their role in providing direct clinical care; this study was driven strongly by nursing leadership. As portal interest screening was incorporated as a required component of the initial admission nursing assessment, this study allowed us to capture sufficient data to understand demographic characteristics associated with portal use.

### Limitations

Nonetheless, there are limitations to nurses alone as the only source of assessing patient interest, with potential bias in interpreting patients’ reasons for *no* interest. In addition, the time of admission may not be the best time during the course of a patient’s hospitalization to make this type of assessment depending on the acuity of a patient’s condition. Although this study did not assess portal enrollment or use, the results highlight potential disparities in a necessary and required upstream process.

### Future Directions

Although Web-based portals have historically been associated with ambulatory care services, interest and research on their use for hospitalized patients are rising. Acute care portals that provide patients with health care information and communication with their teams during hospitalization have been studied for usability [11,12], implementation [13,14], and association with readmission rates [15]. Incorporating acute care portal functionality into a health care system portal may increase interest among hospitalized patients in using a platform that crosses a system’s health care settings, and further research on acute care portal design and implementation is needed. However, our findings suggest that the period of hospitalization also offers an opportunity to engage patients in a health care system portal that lacks acute care functionality. Indeed, after discharge, patients and their loved ones may have greater energy and capacity for understanding the hospital course and discharge instructions; access to this information may facilitate their ability to communicate important information to their ambulatory care providers.

This study is the first to begin to understand issues around portal use among inpatients, specifically in a safety-net setting. Research suggests that vulnerable populations need significant support to register and use a portal website [16,17]. Safety-net hospitals may have significant workflow challenges with providing portal education and support tailored to patients’ language, literacy, vision/hearing, and other needs. These findings triggered local quality improvement initiatives that could be replicated elsewhere: portal and internet education coordinated by the hospital library, in-person and phone technical support for patients, a Web-based curriculum to teach skills in accessing and navigating the portal, and promotional materials about designating a caregiver proxy in our 5 key languages. Future research and policies to incentivize health information technology should incorporate strategies for tailoring patient portal usability and implementation for diverse populations to avoid worsening the digital divide.

## Abbreviations

AOR: adjusted odds ratio
EHR: electronic health record
